# Supplementary material for: Hydrogel Viscoelasticity Modulates Cell Nascent Extracellular Matrix Deposition
Source: Macromol Rapid Commun. 2025 Aug 14;46(21):e00435. doi: 10.1002/marc.202500435 (PMC12590926; doi:10.1002/marc.202500435)
Supplement: Supplementary file 1 — Supporting file: marc70021‐sup‐0001‐SuppMat.pdf. [file MARC-46-e00435-s001.pdf]

Supporting Information

Hydrogel viscoelasticity modulates cell nascent extracellular matrix deposition

*Matthew L. Tan, Avinava Roy, Eleanor M. Plaster, Haguy Wolfenson, Adam Abraham, Claudia Loebel**

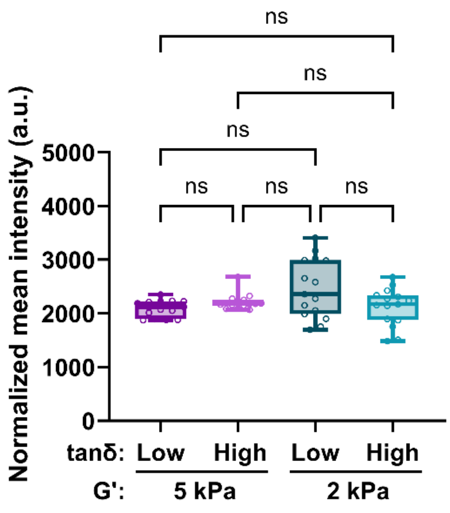

Figure S1. Normalized mean intensity of fluorescent fibronectin adsorbed to hydrogels over 48 h. Data was normalized to background fluorescent intensity of hydrogels incubated without fluorescent fibronectin. N = 5 regions per hydrogel for 3 independent hydrogels. ns = non-significant, Brown-Forsythe and Welch ANOVA with Dunnett T3 test for multiple comparisons.

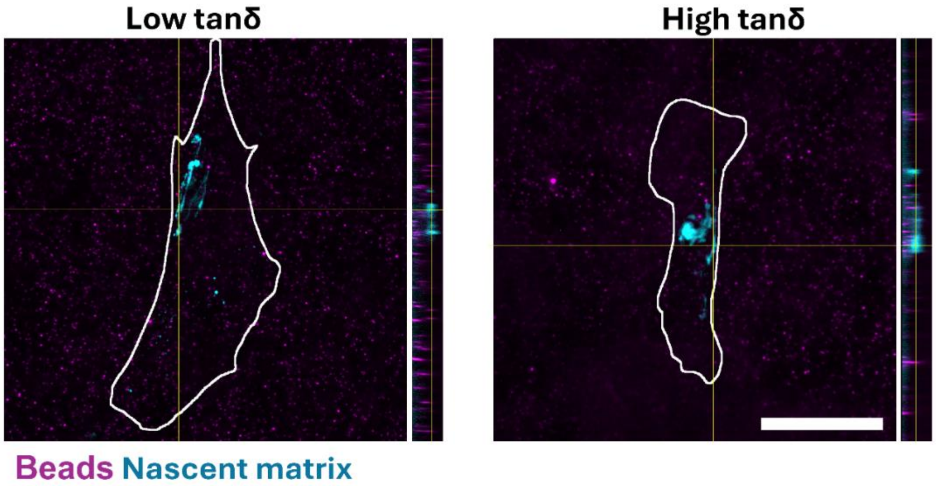

Figure S2. Representative confocal slice and orthogonal projection of cells (cell boundary marked) seeded on bead-laden low and high tanδ hydrogels after 72 h of culture. Scale bar = 50 μm

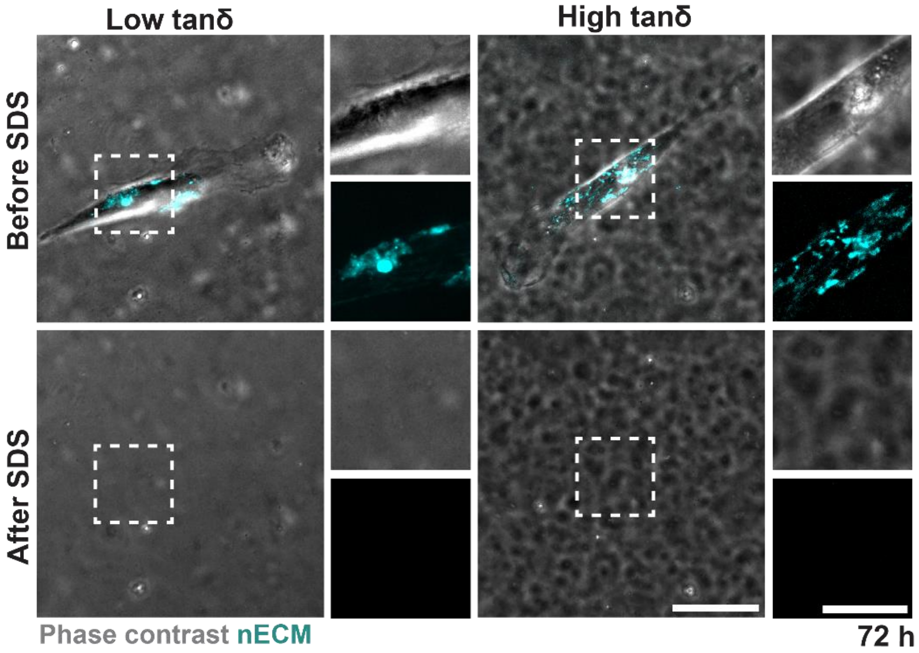

Figure S3. Representative confocal projections of cells and nECM on low and high tanδ hydrogels for 72 h before and after treatment with SDS. Scale bar = 50 μm, 25 μm for insets

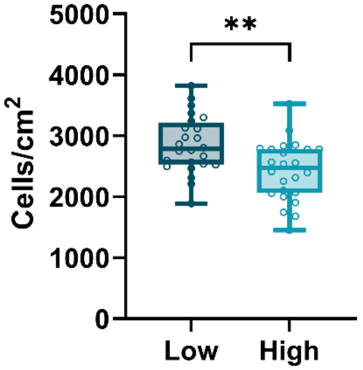

Figure S4. Number of cells per region of interest attached to low and high tanδ hydrogels 3 h post-seeding, wash, and fixation. N = 8 regions per hydrogel for 3 independent hydrogels, **$p<0.01$, two-tailed student's t-test with Welch's correction.

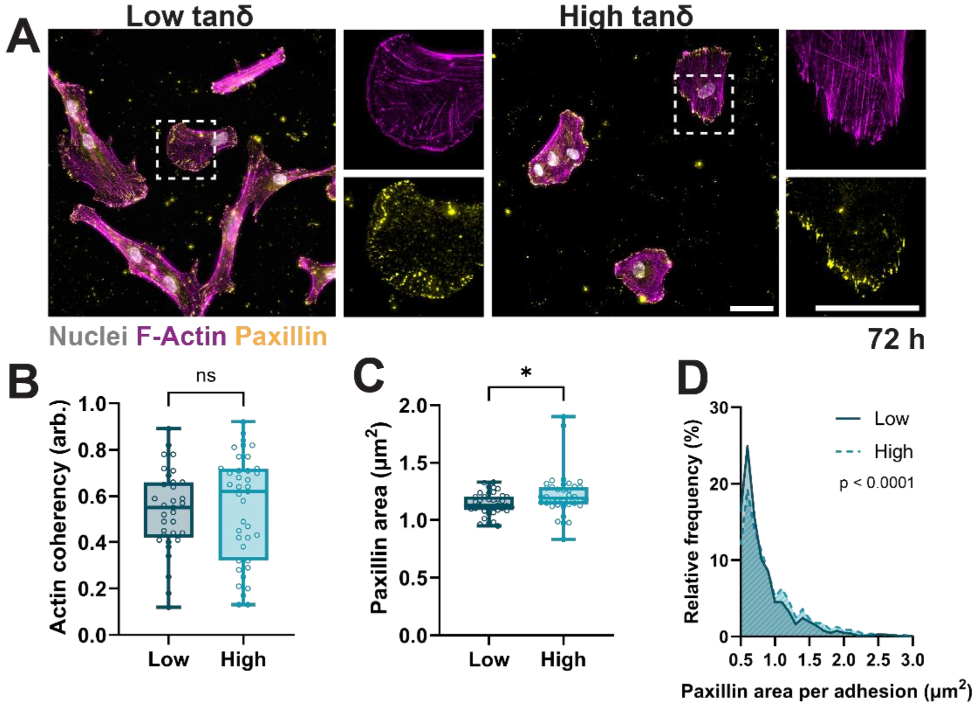

Figure S5. 72 h timepoint of focal adhesion and actin analysis. **A.** Representative fluorescent images of F-Actin and paxillin of hMSCs cultured for 72 h at low and high tanδ hydrogels. Scale bars = 50 µm. **B.** Quantification of actin coherency of hMSCs cultured for 72 h at low and high tanδ hydrogels. N = 33, 39 cells total for low and high tanδ hydrogels respectively, from 3 independent hydrogels. **C.** Quantification of projected paxillin area of hMSCs cultured for 72 h atop low and high tanδ hydrogels. N = 38 cells total from 3 independent hydrogels. **D.** Quantification of relative frequency of single focal adhesion (i.e., paxillin area) per hMSC cultured for 72 h atop low and high tanδ hydrogels. N = 38 cells total from 3 independent hydrogels. ns = non-significant, **p<0.01, ***p<0.001, ***p<0.0001, two-tailed student's t-test with Welch's correction (B,C), chi-square test (D).

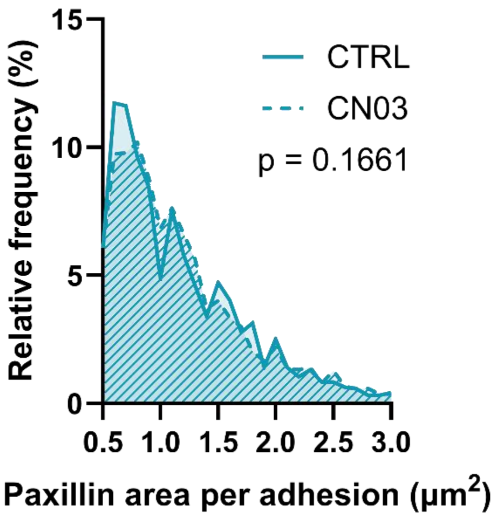

Figure S6: Quantification of relative frequency of single focal adhesion (i.e., paxillin area) per hMSC cultured for 3 h atop non-treated (CTRL) or CN03 treated (CN03) high tanδ hydrogels. N = 36 cells total from 3 independent hydrogels. Chi-square test.

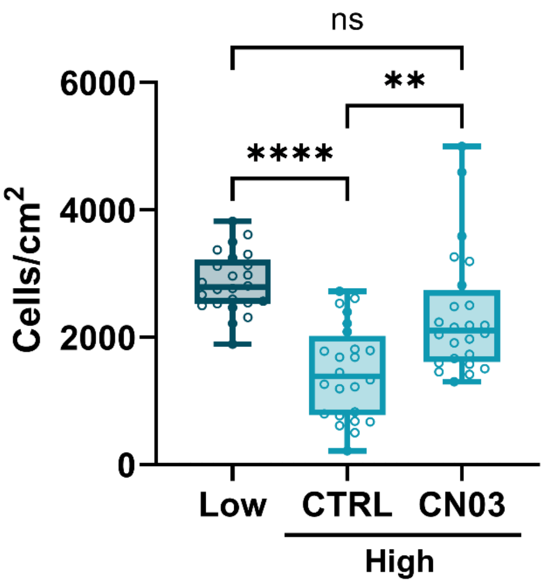

Figure S7. Number of cells per region of interest attached to low tan δ hydrogels without CN03 (Low) and high tanδ hydrogels without (CTRL) or with CN03 treatment (CN03) 3 h post-seeding, wash, and fixation. N = 8 regions per hydrogel for 3 independent hydrogels, ns = non-significant, **p<0.01, ***p<0.001, Brown-Forsythe and Welch ANOVA with Dunnett T3 test for multiple comparisons.

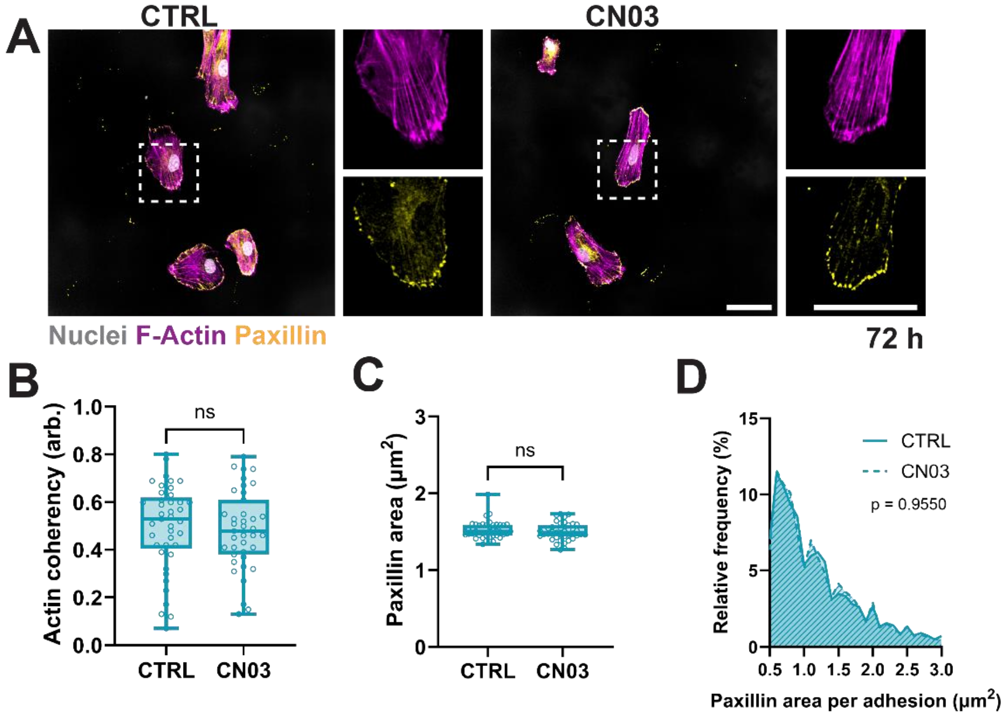

Figure S8. 72 h timepoint of focal adhesion and actin analysis with or without CN03 treatment. **A.** Representative fluorescent images of F-Actin and paxillin of hMSCs cultured for 72 h on high tanδ hydrogels without (CTRL) or with CN03 treatment (CN03). Scale bars = 50 μm. **B.** Quantification of actin coherency of hMSCs cultured for 72 h on high tanδ hydrogels without (CTRL) or with CN03 treatment (CN03). N = 41, 39 cells total for CTRL and CN03 respectively, from 3 independent hydrogels. **C.** Quantification of projected paxillin area of hMSCs cultured for 72 h on high tanδ hydrogels without (CTRL) or with CN03 treatment (CN03). N = 41, 39 cells total for CTRL and CN03 respectively, from 3 independent hydrogels. **D.** Quantification of relative frequency of single focal adhesion (i.e., paxillin area) per hMSC cultured for 72 h atop low and high tanδ hydrogels. N = 41, 39 cells total for CTRL and CN03 respectively, from 3 independent hydrogels. ns = non-significant, two-tailed student's t-test with Welch's correction (B,C), chi-square test (D).
